# Evaluation of safety, effectiveness and reproducibility of telemedicine for neurosurgical screening

**DOI:** 10.31744/einstein_journal/2019AO4609

**Published:** 2019-08-19

**Authors:** Luiz Adriano Esteves, André Tosta Ribeiro, Elton Gomes da Silva, Marcelo Campos Moraes Amato, Leandro Bôa-Hora Rodrigues, Helder Tedeschi, Marcos Juliano dos Santos, Gustav Lebrão, Andrei Fernandes Joaquim

**Affiliations:** 1 Universidade Estadual de Campinas, Campinas, SP, Brazil; 2 Hospital de Força Aérea de São Paulo, São Paulo, SP, Brazil; 3 Hospital Estadual de Franco da Rocha, Franco da Rocha, SP, Brazil

**Keywords:** Neurosurgery, Smartphones/trends, Telemedicine, Neurosurgery/methods, Sciences Health

## Abstract

**Objective:**

To ascertain the safety, effectiveness and reproducibility of screening potential neurosurgical patients by means of smartphones.

**Methods:**

This is a retrospective and multicentric study. Data were collected from the medical records of patients subjected to real emergency neurosurgical evaluations and compared with assessments by neurosurgeons using smartphones to determine the feasibility of identifying changes in cranial computed tomography scans, potentially serious conditions of patients, and the need for transfer to reference centers.

**Results:**

We analyzed 232 cases. The main diagnosis was traumatic brain injury, with 119 cases (51.3%). Of this, 105 (45.3%) patients were discharged immediately after the assessment. The telemedicine evaluators presented 95.69% accuracy in the identification of changes in computed tomography scans, with 0.858 concordance. Accuracy in the identification of severity was 95.26%, with 0.858 concordance. As for procedure, the concordance among evaluators was 0.672, increasing to 100% in cases that required surgical treatment.

**Conclusion:**

Our study indicated that the use of telemedicine for screening patients with acute neurological disorders was safe, effective and reproducible. Implementation of the method shows a promising potential to improve the patient's outcome by reducing unnecessary transfers and decreasing the time elapsed until a specialist can be consulted.

## INTRODUCTION

Throughout the world, including Brazil, requests for neurosurgical evaluations of patients in emergency units are extremely frequent due to the large number of patients suspected of suffering from potentially surgical neurological conditions such as traumatic brain injury (TBI), cerebrovascular accident (CVA), subarachnoid hemorrhage, or infectious and neoplastic diseases.

From the economic and logistical standpoints, the implementation of properly equipped services for neurological care at all emergency units is not feasible. Therefore, patients to be evaluated must be transported to reference centers, where hospital beds must be available. This situation gives rise to two new problems: a delay until care can be provided by a specialist and a delay until a specific treatment can be started, in addition, the unnecessary transfer of the patient.^(^^1^^)^

Telemedicine takes specialized medical care to distant locations, makes it a very useful tool for the health sector, reduces costs and optimizes health care processes, as well as expanding the area of specialized medical coverage.^(^^2^^–^^4^^)^ In the early 21^st^ century, the advent and popularization of the smartphone and the development of broadband data transmission technology allowed reference centers to be connected to more peripheral or geographically distant health service centers, providing immediate assistance to clear up diagnostic doubts and provide advice for treatment protocols.^(^^5^^,^^6^^)^

In this context, it is important to assess the safety and effectiveness of the use of telemedicine as a means of screening patients with possible conditions that require emergency neurosurgical treatment.

## OBJECTIVE

To determine the safety, effectiveness and reproducibility of screening neurological patients using smartphone-assisted telemedicine techniques.

## METHODS

Patients from three neurosurgical reference centers were analyzed: the *Hospital de Força Aérea de São Paulo* (HFASP), in São Paulo (SP), the *Hospital Estadual “Dr. Albano da Franca Rocha Sobrinho”* (HEFR), in Franco da Rocha (SP) and the *Hospital e Maternidade Galileo* (HMG) in Valinhos (SP), Brazil.

The project was submitted and approved by the Institutional Review Board of all the participating institutions and the Institutional Review Board of the *Faculdade de Ciências Médicas* of the *Universidade Estadual de Campinas* (Unicamp), opinion 1.596.946, CAAE: 55907815.9.0000.5404. The informed consent was waived.

### Study design

This survey is part of a study by Esteves^(^^7^^)^ who evaluated the safety and reproducibility of telemedicine employed in neurological emergencies, as an alternative for screening emergency in neurosurgical patients. This is a clinical and retrospective study involving patients evaluated by the neurosurgery team of three institutions. These three hospitals were selected to participate in order to increase the sample by including services with different profiles, thereby obtaining a sample as close as possible to the reality of the state of São Paulo.

A total of 232 patients were evaluated, who had been subjected to cranial computed tomography (CT) scans and to urgent assessment by the neurosurgery team due to suspected neurosurgical disorders from July to September 2016. Patients whose clinical or radiological data were incomplete were excluded.

Information was collected on the history that gave rise to the need for medical care, neurological physical examination, diagnosis, therapy procedures and final outcome. This information was collected from patient records, emergency medical records, and patient referral forms. Only information about the presence or absence of focal neurological *deficits* and pupillary alterations was used in the neurological examination. The neurological status of patients was determined based on the Glasgow Coma Scale (GCS),^(^^8^^)^ while their functional status at the time of hospital discharge was determined using the Glasgow Outcome Scale (GOS).^(^^9^^)^

**Figure 1:**
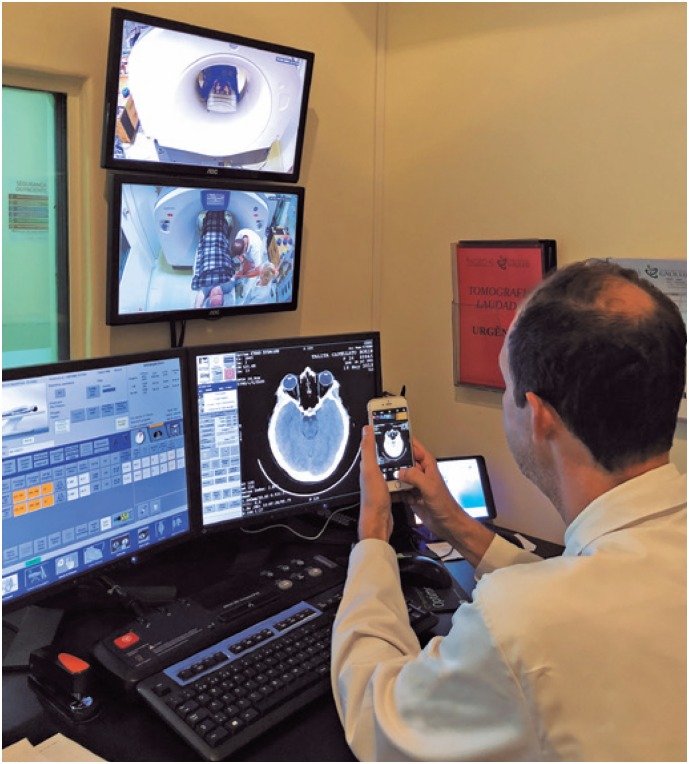
Obtaining computed tomography scan images of the skull

The CT images were obtained using an iPhone 6 smartphone (Apple Inc., Cupertino, CA, USA) to photograph printed exams or film exams displayed on a computer monitor, filmed in 1080p@60fps (Figure 1). Data were stored on hard disk and also placed in cloud storage, using the free Google Drive™ application (Google Inc., Santa Clara, CA, USA).

**Figure 2:**
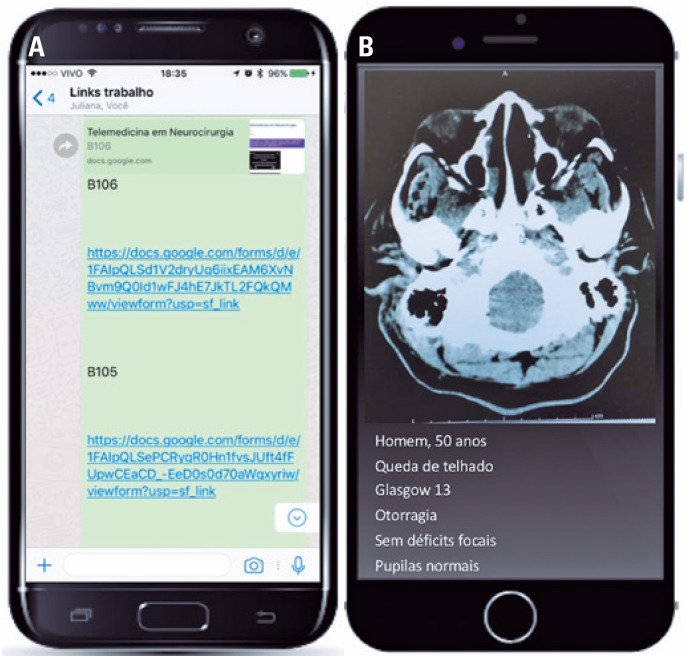
Link sent to reviewers by WhatsApp™ application and case view in the smartphone. (B): Man, 50 years; Fall from roof; Glasgow Coma Scale of 13; Otorrhagia; No focal *deficits*; Normal puplis

**Figure 3:**
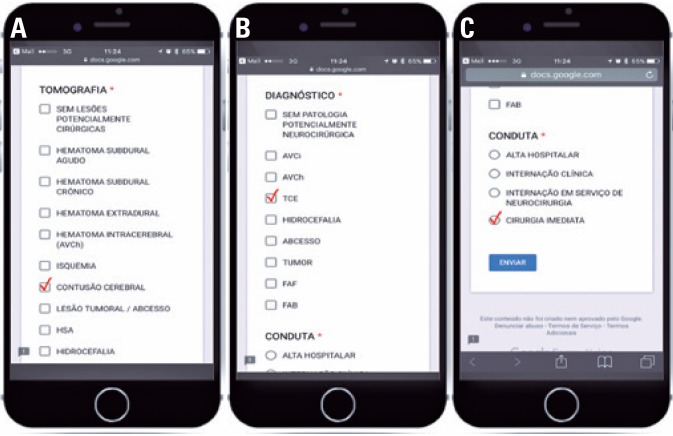
Evaluators response screen. (A) Tomography: No injuries requiring surgery; Acute subdural hematoma; Extradural hematoma; Intracerebral hematoma; Ischemia; Cerebral contusion; Tumor lesion/abcess; SAH; Hydrocephaly; (B) Diagnosis: No diseases requiring neurosurgery; Ischemic stroke; Hemorrhagic stroke; Hydrocephaly; Abcess; Tumor; FAF; FAB; (C) Management: Hospital discharge; Clinical hospitalization; Hospitalization in neurosurgery service; Immediate surgery; Submit

Individual questionnaires were created, one for each case, using the free Google Form™ application (Google Inc., Santa Clara, CA, USA), which generated a link to the form on the Internet). Five independent volunteer evaluators were recruited, who answered a questionnaire for each of the evaluated patients. These evaluators received groups of five links through the WhatsApp™ app for smartphone (Facebook Inc., Menlo Park, CA, USA). Upon accessing the link, the evaluators were directed to a Google Form™ page, in which they answered the questions and sent them to the principal investigator (Figures 2 and 3). The links and other data were sent through a wireless data transmission network equipped with a WPA2-PSK secure data transmission system connected to fixed or mobile broadband Internet with minimum connection speed of 674Kbps.

The questionnaires contained three questions, which were answered after analyzing the cases and CT images on the smartphone: (1) analysis of the cranial CT scan: the evaluator stated whether or not he detected changes in the CT scan; (2) formulation of a diagnostic hypothesis: the evaluator formulated a diagnostic hypothesis of the patient, based on the history and an analysis of the images; (3) procedure: the evaluator selected a procedure for the patient.

The answers to the questions on the questionnaire were sent via smartphone using a 3G or 4G broadband connection.

The reports of the cranial CT scans and the answers of the evaluators were divided into two groups: scans showing no change, and scans showing alterations. The diagnosis of the patients based on their medical records and on the evaluators’ answers was divided into two groups: patient with severe neurological condition, and patient without severe neurological disorder. Conditions classified as severe were based on the following criteria: the presence of changes in the tomographic examination, and the severity score determined by the Advanced Trauma Life Support (ATLS) as moderate or severe (Glasgow ≤12).^(^^10^^)^

Case management was analyzed in three different ways. In the initial analysis, the management options were divided into three groups (discharge, hospitalization, and surgery), in order to compare the procedure recommended by the evaluators with the actual procedure adopted. In this case, inter-rater reliability tests were performed, as well as tests to determine the agreement between raters and patient data. A simulation was performed in which the procedures proposed by the evaluators were divided into two groups: those not requiring hospitalization for neurosurgical intervention, and those requiring hospitalization for neurosurgical intervention. The purpose of this simulation was to analyze inter-rater reliability to determine the need for referral to a neurosurgical service, which is the main point at issue. Lastly, a simulation was performed to broaden the case management options: discharge, hospitalization without need for neurosurgical intervention, hospitalization in a neurosurgical service, and immediate surgery. Again, no concordance test was performed with the real procedure.

The concordance between the two assessments (in person and by telemedicine) was evaluated based on Cohen's Kappa coefficient.^(^^11^^)^ Accuracy, sensitivity and specificity were determined by means of contingency tables.

## RESULTS

Initially, the study involved 232 cases of patients subjected to CT scanning and evaluated by a neurosurgery team between July 1^st^, 2016 and September 30, 2016. These cases were distributed as follows: 56 (24.1%) from the HFASP, 117 (50.4%) from the HEFR and 59 25.4%) from the HMG. The distribution of cases by sex was 141 men and 91 women (1.55:1), and by age it was 10 to 102 years, with an average of 48 years (median of 49).

The main pathology was TBI (51.3%), followed by cases of CVA or stroke (15.1%) and cephalalgia (11.6%).

The time elapsed between the CVA and treatment by the neurosurgery team ranged from 1 to 730 hours, with a mean time of 18 hours, and median time of 5 hours.

The most frequent initial procedure was discharge from hospital after evaluation (105 cases; 45.3%); 101 (43.5%) patients were hospitalized and 26 (11.2%) underwent surgery. The average length of hospitalization was 18.3 days, varying from less than one day to 312 days.

Among the evaluated patients, 27 died (11.7%), and 167 (72%) were discharged with no neurological *deficit*.

### Results of the analysis of computed tomography scans

The evaluators’ answers were compared to each other and to the results of radiology exams, and were divided into two groups: 1 –exam without pathological alterations, and exam with pathological alterations. The main findings are presented in table 1.

**Table 1:** Analysis of changes in computed tomography scan according to the five evaluators (E)

Altered exam	Report	E1	E2	E3	E4	E5
No	145 (62.5)	146 (62.9)	145 (62.5)	148 (63.8)	145 (62.5)	147 (63.4)
Yes	87 (37.5)	86 (37.1)	87 (37.50)	84 (36.0)	87 (37.5)	85 (36.6)
Total	232 (100)	232 (100)	232 (100)	232 (100)	232 (100)	232 (100)

Results expressed as n (%). E: evaluator.

The inter-rater agreement according to Cohen's Kappa coefficient was 0.858 regarding the presence of changes in CT scans, and 0.908 regarding the radiological report, *i.e*., near-perfect concordance. In 194 cases (83.6%), there was concordance between the five evaluators and the CT scan reports. An analysis of the ability to differentiate between normal and altered examinations revealed 93.1% sensitivity, 97.2% specificity and 95.69% accuracy.

### Results of the analysis of diagnosis

The evaluators’ responses and the initial diagnoses proposed in the initial assessment report were divided into two groups: patient without severe neurological pathology, and patient with severe neurological pathology. The main results are described in table 2.

**Table 2:** Distribution of patients according to neurological severity

Severe	Report	E1	E2	E3	E4	E5
No	145 (62.5)	145 (62.5)	144 (62.1)	147 (63.4)	145 (62.5)	147 (63.4)
Yes	87 (37.5)	87 (37.5)	88 (37.9)	85 (36.6)	87 (37.5)	85 (36.6)
Total	232 (100)	232 (100)	232 (100)	232 (100)	232 100)	232 (100)

Results expressed as n (%). E: evaluator.

The degree of concordance between the evaluators regarding the patients’ neurological severity or non-severity was 0.858 according to Cohen's Kappa coefficient, and 0.899 according to the patients’ charts, *i.e*., almost perfect concordance.

In 194 cases (83.6%), there was agreement between the five evaluators and the diagnosis based on the medical record. An analysis of the ability to differentiate between patients with severe neurological impairment and patients without severe neurological impairment revealed 93.1% sensitivity, 96.6% specificity and 95.3% accuracy.

### Results of the analysis of procedures

A comparison was made of the procedures proposed by the evaluators and the ones actually adopted, divided into three groups: hospital discharge, hospitalization, and immediate surgery. The results obtained are described in table 3.

**Table 3:** Procedure adopted and procedure proposed by the evaluators (E)

	Procedure adopted	E1	E2	E3	E4	E5
Hospital discharge	105 (45.3)	91 (39.2)	103 (44.4)	70 (30.2)	70 (30.2)	74 (31.9)
Hospitalization	101 (43.5)	114 (49.1)	101 (43.5)	129 (55.6)	136 (58.6)	124 (53.5)
Immediate surgery	26 (11.2)	27 (11.6)	28 (12.1)	33 (14.2)	26 (11.2)	34 (14.7)
Total	232 (100)	232 (100)	232 (100)	232 (100)	232 (100)	232 (100)

Results expressed as n (%). E: evaluator.

According to Cohen's Kappa statistic, inter-rater concordance was 0.672, which was significant. With respect to the three categories, we obtained a concordance of 0.664 for hospital discharge (significant), 0.613 for hospitalization (significant), and 0.822 for immediate surgery (almost perfect).

Of the 34 patients subjected to surgery, 26 were recommended for surgery at the time of admission and 8 underwent late surgery. In these cases, there was 100% agreement among all the evaluators about the need for immediate surgery or hospitalization in a neurosurgical unit.

In another analysis, the evaluators’ responses were divided into two groups: no need for hospitalization in a neurosurgical unit, and need for hospitalization in a neurosurgical unit (Table 4). The degree of concordance between the evaluators regarding the need or not for referral of patients to a neurosurgical unit, according to Cohen's Kappa statistic, was 0.87, a near perfect concordance.

**Table 4:** Need or no need for hospitalization in a neurosurgical unit

	E1 (%)	E2 (%)	E3 (%)	E4 (%)	E5 (%)
No	161 (69.4)	163 (70.3)	158 (68.1)	161 (69.4)	153 (66.0)
Yes	71 (30.6)	89 (29.7)	74 (31.9)	71 (30.6)	79 (34.1)
Total	232 (100)	232 (100)	232 (100)	232 (100)	232 (100)

Results expressed as n (%). E: evaluator.

Lastly, an analysis was made of the evaluators’ answers divided into four subgroups: hospital discharge, hospitalization without neurosurgery, hospitalization with neurosurgery, and immediate surgery.

According to Cohen's Kappa statistic, the degree of concordance among the evaluators regarding the four procedures was 0.673, which is significant. Concordance regarding immediate surgery was 0.822, *i.e*., near perfect agreement.

## DISCUSSION

In more than half of the cases, the main initial diagnosis was TBI. This finding is consistent with the literature, since TBI is one of the main causes of treatment at emergency units. A 2006 report by the World Health Organization (WHO) estimates that the incidence of TBI with hospitalization in Europe is 235 per 100,000 inhabitants per year.^(^^12^^)^ In the United States the incidence is 538.2 per 100,000 inhabitants.^(^^13^^)^ This is followed by cases of CVA, in which patients do not usually undergo surgical treatment but remain hospitalized, often for lengthy stays. Lastly are cases of cephalalgia, most of which involve patients with minor clinical symptoms who are discharged after neurosurgical assessment.

Several studies suggest that a simplified neurological examination, associated with a CT brain scan, suffices to determine emergency management for neurological patients.^(^^1^^,^^14^^–^^16^^)^ This leads one to question the need for transferring patients for evaluation and for the physical presence of the neurosurgeon for this assessment. The medical literature contains several studies about the use of telemedicine as a screening tool and an aid in decision making in the field of neurosurgery. However, very little research has focused on the use of smartphones as a tool for neurological screening.

In a review of the literature in 2018, Upadhyayula et al.,^(^^17^^)^ state that the time elapsed until treatment and surgery and the absence of trained people are the risk factors with the greatest impact on mortality and unfavorable outcomes in neurosurgery. They conclude that telemedicine consultations to regional neurosurgical centers can potentially shorten the delay in diagnosis and screening, shorten the time to surgery and reduce unnecessary transfers in remote areas.

Chodroff et al.,^(^^18^^)^ reported the results obtained with the implementation of Telemedical Emergency Neurosurgical Network (TENN) in 1992. This project was implemented in California. The system was based on the connection between peripheral hospitals and stations connected through computer networks, with specialist doctors available to clarify doubts. In 3 years, there was a reduction of about 60% in patient transfers for treatment and a reduction in the time elapsed until they underwent surgery. These authors concluded that most patients transferred from peripheral hospitals to specialized neurosurgery evaluation did not require neurosurgical treatment. In addition, an estimated savings of US$ 626,149 was achieved for an investment of US$ 64,375.

A study conducted in 2010 by Klein et al.,^(^^1^^)^ in Israel, evaluated patients with intracerebral hemorrhage treated at hospitals without a neurosurgery team. These patients were divided into three groups. For the first group, neurosurgical services were indispensable. The patients of the second group were evaluated by telemedicine. The patients of the third group were evaluated according to clinical and radiological procedural guidelines. In the second group, 40.9% of patients were transferred to neurosurgery units compared to 74% of patients in the third group. There were no significant differences in outcomes among the three groups. The authors concluded that telemedicine evaluation was safe, reduced the unnecessary transfer of patients to referral services, avoided overcrowding and reduced transportation costs.

In the only study found involving the use of smartphones in Nepal,^(^^19^^)^ 120 patients were evaluated in a prospective study in 2013. Neurosurgery residents photographed the imaging tests of patients and sent them to the head neurosurgeons using a free smartphone application (Viber^®^). The medical procedure proposed by the neurosurgeon who analyzed the data sent by the application was compared with the actual procedure adopted for each case, after a face-to-face assessment. In only 5% of cases was the decision reviewed after the actual evaluation of the images. The authors concluded that the practice of telemedicine via smartphones was useful, but should be employed carefully, since there was a change in procedure in some cases.

It is not possible to make a direct comparison between the discussed papers and our study, because in these papers high definition images were used as well as a discussion with real time experts were performed. Our proposal is to establish minimum necessary conditions for the decision making process, considering information to optimize patients transfer to neurosurgical facilities, using CT scan images and a summarized patient's clinical history. We could infer that if we obtained success with our limited methodological conditions, it would certainly work in a more advanced scenario with better images and a more detailed clinical history.

### Discussion about the methodology of the proposed assessment

#### Reproducibility

The findings described here confirm the reproducibility of the proposed system, since the Cohen's Kappa coefficient was >0.8 in all the analyses of inter-observer concordance pertaining to the interpretation of CT scans, the established diagnosis, and the proposed procedure, indicating almost perfect agreement.

#### Effectiveness

The proposed evaluation system proved to be extremely effective with respect to the three evaluated aspects. The analysis of CT scans revealed an extremely high ability to detect changes in the images, with 95.7% accuracy, 93.1% sensitivity and 97.2% specificity. In one case, a change in an image was found by the evaluators that had gone undetected by the radiologist. The system also showed good performance in establishing a diagnosis of severity, with 95.3% accuracy.

As for case management procedures, the analysis of inter-rater concordance regarding the need or not to send the patient to a neurosurgical unit was 0.87, which is considered a near perfect degree of agreement. In another analysis, the possibilities for hospital discharge, hospitalization and immediate surgery were established, for which an overall concordance of 0.672 was reached, which is significant, and the concordance regarding recommended surgery was almost perfect, *i.e*., 0.822. In the final analysis, which included the possibility of admitting the patient to a service with or without neurosurgery, a significant and almost perfect general concordance for recommended surgery was also obtained. This proves that the application of this system would reduce unnecessary transportation and hospitalization of patients, and also reduce the time elapsed until surgical treatment was applied.

### Safety of the method

In the case in question, the proposed assessment system could not recommend discharge or hospitalization in a healthcare unit without neurosurgical services for a patient that has undergone immediate or delayed surgical treatment. In the 26 cases of immediate surgery, the evaluators did not choose this procedure in only 3 cases, for which they proposed transfer to a hospital with neurosurgical services. In the eight cases of delayed surgery, the evaluators recommended immediate surgery for five of them and hospitalization in a neurosurgery unit for the other cases.

One limitation of our analysis is that patients should be evaluated fully and not only from a neurological perspective. The patient with TBI is, in almost all cases, a patient with systemic trauma with ischemic stroke and who often presents several comorbidities. Another point is that implementing such a model requires complex changes in health care service structures and in the current medical legislation that holds the physician responsible for the decision, even if there has been a discussion with a specialist. It should also be noted that the tools used in our study are not recommended for effective implementation of our model for assistance purposes. For this, it is necessary, due to the current legislation, the use of professional communication platforms that guarantee the confidentiality and security of the information.

## CONCLUSION

The use of telemedicine in our study proved to be safe, effective and reproducible for screening patients with potential neurosurgical disorders.

Our findings demonstrate that the implementation of a screening system similar to the one we used in our study would not only be feasible but would have a significant impact in reducing the number of patient transfers and optimizing patient treatments.

In addition, our study provided subsidies that would make it possible to create a scale, applicable by telemedicine, to classify patients as priority in the transfer and also to debate and improve the legislation, allowing the implementation of conduct through telemedicine.
