# Supplementary material for: Evaluation of safety, effectiveness and reproducibility of telemedicine for neurosurgical screening
Source: Einstein (Sao Paulo). 2019 Aug 19;17(4):eAO4609. doi: 10.31744/einstein_journal/2019AO4609 (PMC6706226; doi:10.31744/einstein_journal/2019AO4609)
Supplement: Translation

# Translation: Avaliação da segurança, da efetividade e da reprodutibilidade na utilização de telemedicina para triagem neurocirúrgica

**Authors:** Luiz Adriano Esteves, André Tosta Ribeiro, Elton Gomes da Silva, Marcelo Campos Moraes Amato, Leandro Bôa-Hora Rodrigues, Helder Tedeschi, Marcos Juliano dos Santos, Gustav Lebrão, Andrei Fernandes Joaquim

**Affiliations:** 1 Universidade Estadual de Campinas, Campinas, SP, Brasil; 2 Hospital de Força Aérea de São Paulo, São Paulo, SP, Brasil; 3 Hospital Estadual de Franco da Rocha, Franco da Rocha, SP, Brasil

**Keywords:** Neurocirurgia, Smartphones/tendências, Telemedicina, Neurocirurgia/métodos, Ciências da Saúde

## Abstract

**Objetivo:**

Verificar a segurança, a efetividade e a reprodutibilidade da triagem de pacientes potencialmente neurocirúrgicos feita por smartphones.

**Métodos:**

Estudo retrospectivo e multicêntrico. Dados de prontuários dos pacientes submetidos à avaliação neurocirúrgica de urgência foram coletados e comparados com a avaliação realizada por neurocirurgiões por meio de smartphones, determinando a capacidade de identificar alterações na tomografia computadorizada de crânio, pacientes potencialmente graves e necessidade de transferência.

**Resultados:**

Foram analisados 232 casos. O principal diagnóstico foi traumatismo crânio encefálico, com 119 casos (51,3%). Destes, 105 (45,3%) tiveram alta após a avaliação. Os avaliadores por telemedicina apresentaram acurácia de 95,69% para identificação de alteração na tomografia computadorizada, com concordância de 0,858. Com relação à identificação de gravidade, a acurácia foi de 95,26%, com concordância de 0,858. Com relação à conduta, os avaliadores apresentaram concordância de 0,672, aumentando para 100% nos casos de tratamento cirúrgico.

**Conclusão:**

O uso da telemedicina em nosso estudo foi seguro, efetivo e reprodutível para triagem dos pacientes com afecções neurológicas agudas. A implementação do método tem potencial de impacto na melhora do resultado para o paciente devido à redução das transferências desnecessárias e do tempo até o atendimento.

## INTRODUÇÃO

Em esfera global, incluindo o Brasil, as solicitações de avaliações neurocirúrgicas dos pacientes em unidades de emergência são extremamente frequentes, devido ao grande número de pacientes apresentando suspeitas de condições neurológicas com potencial cirúrgico, como traumatismo craniencefálico (TCE), acidente vascular cerebral (AVC), hemorragia subaracnoide e/ou doenças infecciosas e neoplásicas.

Do ponto de vista econômico e logístico, a implementação de serviços devidamente equipados para assistência neurológica em todas as unidades de emergência não é viável. Assim, pacientes que requerem avaliação devem ser transportados para centros de referência dispondo de leitos hospitalares. Desta situação, originam-se dois novos problemas: aguardar até que o atendimento seja prestado por um especialista e esperar até que um tratamento específico possa ser iniciado, além da transferência/remoção desnecessária do paciente.^(^^1^^)^

A telemedicina tem possibilitado atendimento médico especializado, mesmo em locais distantes. Além disso, trata-se de ferramenta muito útil para o setor de saúde, que promove a redução de custos, otimiza os processos de assistência à saúde e oferta à área atendimento médico especializado.^(^^2^^–^^4^^)^ No início do século 21, o advento e a popularização dos smartphones, e o desenvolvimento da tecnologia de transmissão de dados em banda larga permitiram que os centros de referência estivessem conectados aos centros de saúde mais periféricos ou geograficamente distantes. Tal tecnologia ainda fornece assistência imediata para esclarecer dúvidas diagnósticas e aconselhar os protocolos de tratamento.^(^^5^^,^^6^^)^

Nesse contexto, é pertinente avaliar a segurança e a efetividade do uso da telemedicina como forma de triagem de pacientes com possíveis condições que necessitem de tratamento neurocirúrgico de emergência.

## OBJETIVO

Verificar a segurança, a eficácia e a reprodutibilidade da triagem de pacientes neurológicos por técnicas de telemedicina assistida por smartphones.

## METÓDOS

A análise incluiu pacientes de três centros cirúrgicos de referência dos seguintes hospitais: Hospital de Força Aérea de São Paulo (HFASP) em São Paulo (SP), Hospital Estadual “Dr. Albano da Franca Rocha Sobrinho” (HEFR) em Franco da Rocha (SP), e Hospital e Maternidade Galileo (HMG) em Valinhos (SP).

O projeto foi submetido e aprovado pelo Comitê de Ética institucional de todas as instituições participantes, e ao Comitê de Ética e Pesquisa da Faculdade de Ciências Médicas da Universidade Estadual de Campinas (Unicamp), parecer 1.596.946, CAAE: 55907815.9.0000.5404. Não houve necessidade do Termo de Consentimento devido ao caráter do estudo.

### Desenho do estudo

Esta pesquisa é parte do estudo conduzido por Esteves,^(^^7^^)^ que avaliaram a segurança e a reprodutibilidade de aplicar a telemedicina em situações de emergência neurológica como alternativa para a triagem de emergência de pacientes neurocirúrgicos. Apresentamos estudo clínico e retrospectivo envolvendo pacientes avaliados pela equipe de neurocirurgia de três instituições de saúde. Foram selecionados três hospitais a fim de aumentar a amostra e incluir serviços com diferentes perfis e obter amostragem o mais próxima possível da realidade do Estado de São Paulo.

Foram avaliados 232 pacientes submetidos à tomografia computadorizada (TC) de crânio e avaliação urgente pela equipe de neurocirurgia, devido à suspeita de distúrbios neurocirúrgicos, no período de julho a setembro de 2016. Foram excluídos os pacientes com dados clínicos ou radiológicos incompletos.

Foram coletadas informações referente ao histórico prévio que originou a necessidade do atendimento médico, ao exame físico neurológico, ao diagnóstico, a procedimentos terapêuticos e ao resultado final. Esta informação foi coletada a partir de registros de pacientes, registros médicos de emergência e formulários de encaminhamento de pacientes. Apenas informações sobre a presença ou ausência de *deficits* neurológicos focais e alterações pupilares foram utilizadas no exame neurológico. O estado neurológico dos pacientes foi estabelecido com base na Escala de Coma de Glasgow (ECG),^(^^8^^)^ enquanto o estado funcional na alta hospitalar foi determinado por meio da Escala de Resultados de Glasgow (ERG).^(^^9^^)^

As imagens de TC foram obtidas por meio de smartphone iPhone 6 (Apple Inc., Cupertino, CA, USA) para fotografar exames impressos ou filmar exames disponíveis em monitores de computadores, filmados em 1080p@60fps (Figura 1). Os dados foram armazenados em disco rígido e também salvos na nuvem, utilizando o aplicativo gratuito Google Drive™ (Google Inc., Santa Clara, CA, USA).

**Figura 1:** Obtenção das imagens da tomografia computadorizada do crânio

Foram criados questionários individuais, um para cada caso, por meio do recurso gratuito Google Form™ (Google Inc., Santa Clara, Califórnia, EUA), que gerou um link para o formulário na Internet). Cinco avaliadores independentes e voluntários responderam a questionário para cada um dos pacientes avaliados. Esses avaliadores receberam um conjunto de cinco links por meio do aplicativo WhatsApp™ para smartphone (Facebook Inc., Menlo Park, CA, EUA). Ao acessar o link, os avaliadores eram direcionados para uma página do Google Form™, onde respondiam as perguntas e as enviavam ao investigador principal (Figuras 2 e 3). Os links e outros dados foram enviados por meio de uma rede de transmissão de dados sem fio equipada com um sistema seguro de transmissão de dados WPA2-PSK conectado à Internet de banda larga fixa ou móvel, com velocidade de conexão mínima de 674Kbps.

**Figura 2:** Link enviado aos revisores via WhatsApp™ e visualização do caso no smartphone

**Figura 3:** Tela de resposta dos avaliadores

Os questionários continham três questões que foram respondidas, após análise dos casos e das imagens de TC no smartphone: (1) análise da TC do crânio: o avaliador afirmou se detectou ou não as alterações na TC (2) formulação de uma hipótese diagnóstica: o avaliador formulou uma hipótese diagnóstica do paciente, baseada na histórico e na análise das imagens; (3) procedimento: o avaliador selecionou um procedimento para o paciente.

As respostas às perguntas do questionário foram enviadas via smartphone com uso de conexão de banda larga 3G ou 4G.

Os relatos das imagens de TC craniana e as respostas aos avaliadores foram divididas em dois grupos: varredura sem alteração e varredura mostrando alterações. O diagnóstico dos pacientes com base em seus prontuários e nas respostas dos avaliadores foi dividido em dois grupos: paciente com quadro neurológico grave e paciente sem distúrbio neurológico grave. As condições classificadas como graves foram baseadas nos seguintes critérios: presença de alterações no exame tomográfico e escore de gravidade determinado pelo Suporte Avançado de Vida em Trauma (ATLS - *Advanced Trauma Life Support*) como moderado ou grave (Glasgow ≤12).^(^^10^^)^

O gerenciamento dos casos foi analisado de três modos distintos. Na análise inicial, por meio do gerenciamento das opções, foram divididos em três grupos (alta hospitalar, hospitalização e cirurgia), para comparar o procedimento recomendado por avaliadores com procedimentos atuais adotados. Neste caso, os testes foram realizados também como determinado, com base no acordo entre variações e dados dos pacientes. Realizou-se simulação com a divisão dos procedimentos propostos pelos avaliadores em dois grupos: não requer hospitalização por intervenção neurocirúrgica e requer hospitalização por intervenção neurocirúrgica. O objetivo desta simulação foi analisar a confiabilidade entre avaliadores para determinar a necessidade de encaminhamento para um serviço de neurocirurgia, que é o ponto principal em avaliação. Por fim, foi conduzida simulação para ampliar as opções de tratamento dos casos: alta, hospitalização sem necessidade de intervenção neurocirúrgica, internação em serviço neurocirúrgico e cirurgia imediata. Novamente, nenhum teste de concordância foi realizado com o procedimento real.

A concordância entre as duas avaliações (pessoalmente e por telemedicina) foi avaliada com base no coeficiente Kappa de Cohen.^(^^11^^)^ A acurácia, a sensibilidade e a especificidade foram determinadas com uso de tabelas de contingência.

## RESULTADOS

Inicialmente, o estudo envolveu 232 pacientes sujeitos à imagem de TC e avaliados por equipe de neurocirurgia entre 1° de julho de 2016 e 30 de setembro de 2016. Esses casos foram distribuídos da seguinte maneira: 56 (24,1%) do HFASP, 117 (50,4%) do HEFR, 59 (25,4%) do HMG. A distribuição dos casos por sexo foi 141 homens e 91 mulheres (1,55:1), e idade 10 a 102 anos, com média de 48 anos (média de 49 anos).

A principal patologia foi TCE (51,3%), seguida por casos de AVC (15,1%) e cefaleia (11,6%).

O tempo decorrido entre o AVC e o tratamento pela equipe de neurocirurgia variou de 1 a 730 horas, com média de 18 horas e de 5 horas.

O procedimento inicial mais frequente foi alta hospitalar após avaliação (105 casos; 45,3%); 101 (43,5%) pacientes foram hospitalizados e 26 (11,2%) submetidos à cirurgia. O tempo médio de internação foi de 18,3 dias, variando entre menos de 1 a 312 dias.

Entre os pacientes avaliados, 27 foram a óbito (11,7%) e 167 (72%) tiveram alta sem *deficit* neurológico.

### Resultados das análises das imagens por tomografia computadorizada

As respostas dos avaliadores foram comparadas entre si, e os resultados dos exames radiológicos foram divididos em dois grupos: exame sem alterações patológicas e exame com alterações patológicas. A tabela 1 descreve os principais achados.

**Tabela 1:** Análise das mudanças no exame de tomografia computadorizada com base na resposta dos cinco avaliadores (A)

Alteração no exame	Relatório	A1	A2	A3	A4	A5
Não	145 (62,5)	146 (62,9)	145 (62,5)	148 (63,8)	145 (62,5)	147 (63,4)
Sim	87 (37,5)	86 (37,1)	87 (37,50)	84 (36,0)	87 (37,5)	85 (36,6)
Total	232 (100)	232 (100)	232 (100)	232 (100)	232 (100)	232 (100)

Resultados expressos como n (%). A: avaliador.

A concordância entre os avaliadores, de acordo com o coeficiente de Kappa de Cohen, foi de 0,858, em relação à presença de alterações nas tomografias, e 0,908 em relação ao relatório radiológico, ou seja, concordância quase perfeita. Em 194 casos (83,6%), observou-se concordância entre os cinco avaliadores e os laudos da TC. Uma análise da habilidade de diferenciação entre exames normais e alterados revelaram sensibilidade de 93,1%, 97,2% de especificidade e 95,69% de acurácia.

### Resultados das análises dos diagnósticos

A avaliação das respostas e o diagnóstico inicial proposto no relatório de avaliação inicial foram dividos em dois grupos: paciente sem lesão neurológica grave e paciente com doenças neurológica grave. Os resultados estão na tabela 2.

**Tabela 2:** Distribuição de paciente conforme gravidade neurológica

Gravidade	Relatório	A1	A2	A3	A4	A5
Não	145 (62,5)	145 (62,5)	144 (62,1)	147 (63,4)	145 (62,5)	147 (63,4)
Sim	87 (37,5)	87 (37,5)	88 (37,9)	85 (36,6)	87 (37,5)	85 (36,6)
Total	232 (100)	232 (100)	232 (100)	232 (100)	232 100)	232 (100)

Resultados expressos como n (%). A: avaliador.

O grau de concordância entre os avaliadores em relação a gravidade e não gravidade neurológicas do paciente foi 0,858 de acordo com coeficiente Kappa de Cohen, e 0,899 de acordo com quadros do paciente, ou seja, concordância quase perfeita.

Em 194 casos (83,6%), observou-se concordância entre os cinco avaliadores e os laudos da TC. Análise de habilidade para diferenciação entre pacientes com lesão neurológica grave e pacientes sem lesão revelou 93,1 de sensibilidade, 96,9% de especificidade e 95,3% de acurácia.

### Resultados das análises dos procedimentos

Foram comparados os procedimentos propostos pelos avaliadores e os que foram efetivamente adotados, divididos em três grupos: alta hospitalar, internação e cirurgia imediata. Os resultados obtidos estão descritos na tabela 3.

**Tabela 3:** Procedimento adotado em procedimentos propostos pelos avaliadores (A)

	Procedimento adotado	A1	A2	A3	A4	A5
Alta hospitalar	105 (45,3)	91 (39,2)	103 (44,4)	70 (30,2)	70 (30,2)	74 (31,9)
Hospitalização	101 (43,5)	114 (49,1)	101 (43,5)	129 (55,6)	136 (58,6)	124 (53,5)
Cirúrgia imediata	26 (11,2)	27 (11,6)	28 (12,1)	33 (14,2)	26 (11,2)	34 (14,7)
Total	232 (100)	232 (100)	232 (100)	232 (100)	232 (100)	232 (100)

Resultados expressos como n (%). A: avaliador.

De acordo com a estatística Kappa de Cohen, a concordância entre avaliadores foi de 0,672, o que foi significativo. Com relação às três categorias, obtivemos concordância de 0,664 para alta hospitalar (significativa), 0,613 para internação (significativa) e 0,822 para cirurgia imediata (quase perfeita).

Dos 34 pacientes submetidos à cirurgia, 26 foram recomendados para cirurgia no momento da internação e 8 foram submetidos à cirurgia tardia. Nesses casos, houve 100% de concordância entre todos os avaliadores sobre a necessidade de cirurgia imediata ou internação em uma unidade de neurocirurgia.

Em outra análise, as respostas dos avaliadores foram divididas em dois grupos: sem necessidade de internação em uma unidade neurocirúrgica e com necessidade de internação em uma unidade neurocirúrgica (Tabela 4). O grau de concordância entre os avaliadores em relação à necessidade ou não de encaminhamento de pacientes para uma unidade neurocirúrgica, segundo a estatística Kappa de Cohen, foi de 0,87 − concordância esta quase perfeita.

**Tabela 4:** Necessidade ou não de hospitalização em unidade neurocirúrgica

	A1	A2	A3	A4	A5
Não	161 (69,4)	163 (70,3)	158 (68,1)	161 (69,4)	153 (66,0)
Sim	71 (30,6)	89 (29,7)	74 (31,9)	71 (30,6)	79 (34,1)
Total	232 (100)	232 (100)	232 (100)	232 (100)	232 (100)

Resultados expressos como n (%). A: avaliador.

Por fim, foi feita análise das respostas dos avaliadores divididos em quatro subgrupos: alta hospitalar, hospitalização sem neurocirurgia, internação com neurocirurgia e cirurgia imediata.

Segundo a estatística Kappa de Cohen, o grau de concordância entre os avaliadores em relação aos quatro procedimentos foi de 0,673, o que é significativo. A concordância em relação à cirurgia imediata foi de 0,822, ou seja, concordância quase perfeita.

## DISCUSSÃO

Em mais da metade dos casos, o principal diagnóstico inicial foi a TCE. Esse achado foi consistente com a literatura, uma vez que a TCE é uma das principais causas de tratamento nas unidades de emergência. Um relatório de 2006 da Organização Mundial da Saúde (OMS) estima que a incidência de TCE com hospitalização na Europa é de 235 por 100 mil habitantes/ano.^(^^12^^)^ Nos Estados Unidos, a incidência é de 538,2 por 100 mil habitantes.^(^^13^^)^ Essa incidência é seguida dos casos de AVC, nos quais os pacientes geralmente não são submetidos ao tratamento cirúrgico, mas permanecem internados − muitas vezes por longos períodos. Por último, estão os casos de cefaleia; a maioria deles envolve pacientes com sintomas clínicos menores, que recebem alta após avaliação neurocirúrgica.

Diversos estudos sugerem que exame neurológico simplificado associado a a exame de TC é suficiente para determinar o manejo de emergência de pacientes neurológicos.^(^^1^^,^^14^^–^^16^^)^ Tais achados nos leva a questionar a necessidade de transferir pacientes para avaliação e questões relacionadas a presença física do neurocirurgião para essa avaliação. A literatura médica contém vários estudos relacionados ao uso da telemedicina como ferramenta de triagem e para apoio da tomada de decisão no campo da neurocirurgia. Contudo, são escassos os estudos que focam no uso de smartphones como ferramenta para triagem neurológica.

Em revisão da literatura em 2018, Upadhyayula et al.,^(^^17^^)^ estabeleceram o tempo envolvido entre o tratamento e cirurgia, e a ausência de treinamento de pessoal, que são fatores de risco com aumento do impacto na mortalidade e nos resultados em neurocirurgia. Concluiu-se que as consultas em telemedicina para centros de neurocirurgia regional podem diminuir potencialmente o atraso no diagnóstico e na triagem, e reduzir o tempo de cirurgia e as transferências desnecessárias em áreas remotas.

Chodroff et al.,^(^^18^^)^ relataram os resultados obtidos com a implantação da *Telemedical Emergency Neurosurgical Network* (TENN) em 1992, em projeto implementado na Califórnia. O sistema foi baseado na conexão entre hospitais periféricos e estações conectadas por redes de computadores, com médicos especialistas disponíveis para esclarecer dúvidas. Em 3 anos, houve redução de cerca de 60% das transferências de pacientes para tratamento e redução do tempo até a cirurgia. Os autores concluíram que a maioria dos pacientes transferidos de hospitais periféricos para avaliação especializada em neurocirurgia não necessitava de tratamento neurocirúrgico. Além disso, possibilitou economia de US$ 626,149 alcançada com investimento de US$ 64,375.

Um estudo conduzido em 2010 em Israel por Klein et al.,^(^^1^^)^ avaliou pacientes com hemorragia intracerebral tratados em hospitais sem equipe de neurocirurgia. Esses pacientes foram divididos em três grupos. Para o primeiro grupo, os serviços neurocirúrgicos eram indispensáveis. Os pacientes do segundo grupo foram avaliados por telemedicina. Os pacientes do terceiro grupo foram avaliados com base nas diretrizes clínicas e radiológicas dos procedimentos. No segundo grupo, 40,9% dos pacientes foram transferidos para unidades de neurocirurgia, em comparação com 74% dos pacientes no terceiro grupo. Não houve diferenças significativas nos resultados entre os três grupos. Os autores concluíram que a avaliação da telemedicina era segura, reduzindo a transferência desnecessária dos pacientes para os serviços de referência, evitando a superlotação e reduzindo os custos de transporte.

No único trabalho encontrado envolvendo o uso de smartphones,^(^^19^^)^ realizado no Nepal, foram avaliados 120 pacientes em um estudo prospectivo conduzido em 2013. Os residentes de neurocirurgia fotografaram os exames de imagem de pacientes e os enviaram aos neurocirurgiões-chefes usando um aplicativo gratuito para smartphone (Viber®). O procedimento médico proposto pelo neurocirurgião, que analisou os dados enviados pelo aplicativo, foi comparado com o procedimento adotado para cada caso, após avaliação face a face. Em apenas 5% dos casos a decisão foi revisada após a avaliação real das imagens. Os autores concluíram que a prática da telemedicina via smartphones foi útil, mas que deve ser empregada com cautela, uma vez que houve mudança de procedimento em alguns casos.

Não é possível fazer comparação direta entre os artigos discutidos e nosso estudo, pois, nesses artigos, foram utilizadas imagens de alta definição, bem como discussão com especialistas em tempo real. Nossa proposta é estabelecer condições mínimas necessárias para o processo de tomada de decisão, considerando informações que poderiam otimizar a transferência de pacientes para instalações de neurocirurgia, usando imagens de TC e histórico clínico resumido do paciente. Portanto, é possível inferir que, se obtivéssemos sucesso com nossas condições metodológicas limitadas, certamente a proposta teria resultado positivo em um cenário mais avançado, com imagens melhores e um histórico clínico mais detalhado.

### Discussão sobre a metodologia da avaliação proposta

#### Reprodutibilidade

Os achados aqui descritos confirmam a reprodutibilidade do sistema proposto, uma vez que o coeficiente Kappa de Cohen foi >0,8 em todas as análises de concordância interobservadores referentes à interpretação de TC, ao diagnóstico estabelecido e ao procedimento proposto, o que indicou concordância quase perfeita.

#### Eficiência

O sistema de avaliação proposto mostrou-se extremamente eficaz em relação aos três aspectos avaliados. A análise das tomografias revelou capacidade extremamente alta de detectar alterações nas imagens, com 95,7% de acurácia, 93,1% de sensibilidade e 97,2% de especificidade. Em um caso, foi apontada mudança na imagem pelos avaliadores que não foi detectada pelo radiologista. O sistema também mostrou bom desempenho no estabelecimento do diagnóstico de gravidade, com 95,3% de acerto.

Quanto aos procedimentos de gerenciamento dos casos, a análise da concordância interavaliadores quanto à necessidade ou não de encaminhar o paciente para uma unidade de neurocirurgia foi de 0,87, o que é considerado um grau de concordância quase perfeito. Em outra análise, estabeleceram-se as possibilidades de alta hospitalar, internação e cirurgia imediata, para as quais foi alcançada concordância global de 0,672, o que é significativo, e a concordância quanto à cirurgia recomendada foi quase perfeita (0,822). Em última análise, que incluiu a possibilidade de admitir o paciente a um serviço com ou sem neurocirurgia, também foi obtida concordância geral significativa e quase perfeita para a cirurgia recomendada. Isso prova que a aplicação desse sistema reduziria o transporte desnecessário e a hospitalização dos pacientes, além de reduzir o tempo percorrido até a aplicação do tratamento cirúrgico.

#### Segurança do método

No caso em questão, o sistema de avaliação proposto não poderia recomendar a alta hospitalar ou a internação em uma unidade de saúde sem serviços neurocirúrgicos para paciente submetido ao tratamento cirúrgico imediato ou tardio. Nos 26 casos de cirurgia imediata, os avaliadores não escolheram esse procedimento em apenas 3 casos, para os quais propuseram a transferência para um hospital com serviços neurocirúrgicos. Nos oito casos de cirurgia tardia, os avaliadores recomendaram a cirurgia imediata para cinco deles e a internação em uma unidade de neurocirurgia para os demais casos.

Uma limitação de nossa análise é a necessidade de os pacientes serem avaliados de forma completa, e não apenas do ponto de vista neurológico. O paciente com TCE é, em quase todos os casos, um paciente com trauma sistêmico, enquanto um paciente com acidente vascular cerebral isquêmico geralmente apresenta várias comorbidades. Outro ponto é a implementação de tal modelo, que requer mudanças complexas nas estruturas dos serviços de saúde e na legislação médica atual, que responsabiliza o médico pela decisão, que é discutida com especialista. Deve-se notar que as ferramentas utilizadas em nosso estudo não são recomendadas para a implementação efetiva em modelos para fins assistenciais. Para tanto, é necessário, devido à legislação vigente, utilizar plataformas de comunicação profissional que garantam a confidencialidade e a segurança das informações.

## CONCLUSÃO

O uso da telemedicina em nosso estudo mostrou-se seguro, eficaz e reprodutível para a triagem de pacientes com possíveis transtornos neurocirúrgicos.

Nossos resultados demonstram que a implementação de um sistema de triagem semelhante ao que usamos em nosso estudo não seria apenas viável, mas impactaria significativamente na redução do número de transferências de pacientes e na otimização dos tratamentos.

Além disso, nosso estudo fornece subsídios que possibilitariam a criação de uma escala, aplicável por telemedicina, para classificar os pacientes como prioritários na transferência e também no debate e melhora da legislação, permitindo a implementação de conduta médicas por meio da telemedicina.
